# Peer Review of “Evaluating the Financial Factors Influencing Maternal, Newborn, and Child Health in Africa: Tobit Regression and Data Envelopment Analysis”

**DOI:** 10.2196/85382

**Published:** 2025-11-28

**Authors:** Titilayo Deborah Olorunyomi

**Keywords:** financial determinants, maternal, newborn, and child health, health care efficiency, Africa, health expenditure, data envelopment analysis, Tobit regression

*This is the peer-review report for “Evaluating the Financial Factors Influencing Maternal, Newborn, and Child Health in Africa: Tobit Regression and Data Envelopment Analysis.”*

## Round 1 Review

The manuscript [1] provides a valuable contribution to the understanding of health care system efficiency in Africa, particularly in the context of maternal, newborn, and child health. The study is ethical, with appropriate use of data from reputable sources such as the World Health Organization, and the methods employed—data envelopment analysis and Tobit regression—are suitable for assessing the technical efficiency of health care systems across 46 African countries.

The material is original, and the paper addresses a significant gap in the literature by focusing on the financial and efficiency factors impacting maternal, newborn, and child health in Africa. Related work is discussed and cited adequately, although a few more recent studies could be included to strengthen the literature review.

The writing is generally clear, though there are some areas where the discussion of the results could benefit from more detail. The study methods are appropriate for the research objectives, and the data used appear to be valid and reliable. The findings are significant and present actionable insights for policymakers, especially in terms of understanding the inefficiencies in the health care systems that impact maternal, newborn, and child health outcomes.

The conclusions are reasonable and are supported by the data, although more detailed recommendations for practical application could enhance the paper’s impact. The topic is certainly of interest to the readership, as it addresses key issues surrounding health care efficiency and the achievement of Sustainable Development Goal 3 in Africa.

Overall, I recommend the manuscript for publication with minor revisions to improve the clarity of some sections and provide more detailed policy recommendations.
